# Squamous cell carcinoma of the conjunctiva

**Published:** 2017-02-10

**Authors:** Stephen Gichuhi, Mandeep S Sagoo

**Affiliations:** 1 Consultant Ophthalmologist and Senior Lecturer: Department of Ophthalmology, University of Nairobi, Kenya.; 2 Consultant Ophthalmic Surgeon: Ocular Oncology Service, Moorfields Eye Hospital and Senior Lecturer: UCL Institute of Ophthalmology, London, UK.

## Introduction and epidemiology

Squamous cell carcinoma of the conjunctiva is the end-stage of a spectrum of disease referred to as ocular surface squamous neoplasia (OSSN). OSSN is a malignant disease of the eyes that can lead to loss of vision and, in severe cases, death. The main risk factors for both are exposure to solar ultraviolet radiation outdoors, HIV/AIDS, human papilloma virus and allergic conjunctivitis. The limbal epithelial cells appear to be the progenitorsof this disease.

OSSN is an important ophthalmic public health problem in equatorial Africa, where there are both high levels of UV radiation and a high incidence of HIV/AIDS. Africa has the highest incidence of OSSN in the world, affecting about 1.3 people per 100,000 population per year; so, if you work in an eye clinic serving a population of 1 million people, you could expect to see one case each month if they all came to the clinic.^1^ By contrast, the incidence in other regions is about 0.1 people per 100,000 population per year, over 10 times lower.

Two disease patterns occur. In equatorial Africa, OSSN affects younger adults and proportionally more women than in other parts of the world. Recent studies in Kenya, for example, found that the mean age of OSSN patients is around 40 years, two-thirds are women and about three-quarters are living with HIV. Elsewhere, OSSN affects older adults (the mean age is about 60 years) and 70% are male.

## Clinical presentation

**Figure 1.**
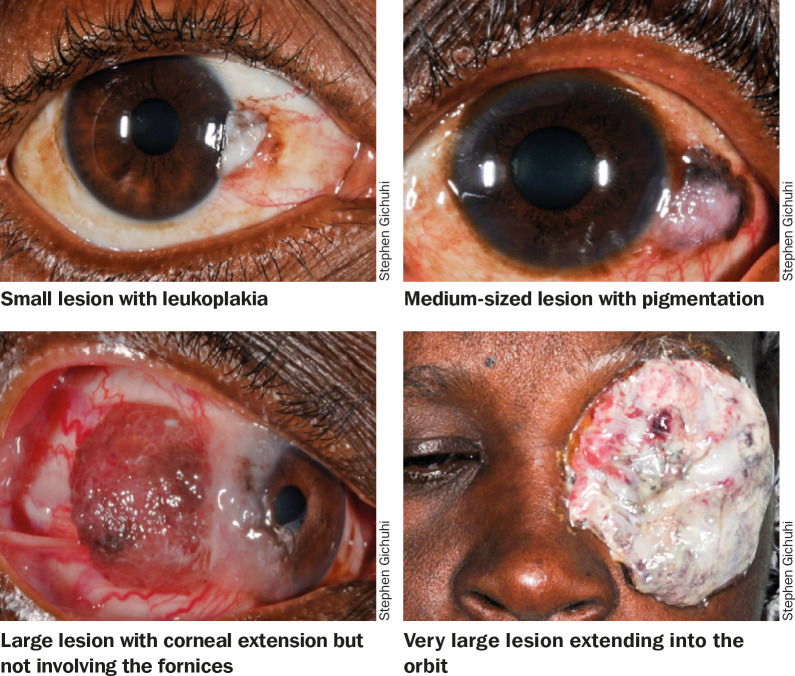
A range of OSSN presentations seen in East Africa.^1^

This disease has a variable appearance (Figure 1). Red eye, photophobia, irritation, foreign body sensation and a white, painless, progressive growth on the surface of the eye are common presenting symptoms.^2^ Most lesions occur in the interpalpebral fissure, especially on the nasal side.^3^ They involve the conjunctiva and may extend onto the peripheral cornea, so visual acuity is often normal in the early stages. It usually only involves one eye. The surface may be gelatinous, papillomatous or fibro-vascular. There is usually inflammation, leukoplakia and markedly dilated blood vessels, referred to as feeder vessels. Some brown to black pigmentation of the lesion is common in African population groups. Most lesions are about 7 mm wide at presentation but late presentation with large orbital tumours are not uncommon.

## Diagnosis

Most cases are diagnosed from the clinical impression. There is a shortage of histopathology services in most equatorial countries; however, even in countries without this limitation, about half of the lesions are not excised for histopathology. This may be related to the increasing trend to treat these lesions with primary topical medication. However, the clinical impression is unreliable, especially in equatorial Africa, as both benign and malignant lesions have overlapping features. There is also the ethical consideration of using potentially dangerous topical medications, such as cytotoxic drugs, without a tissue diagnosis.

Histopathology is the gold standard for diagnosis: the pathologist will see an abrupt transition between the normal and abnormal tissues. However, histopathology is not without challenges. It requires surgical intervention for excision and the interpretation is subjective, varying between pathologists. It is particularly challenging in the earlier stages of OSSN, when it is pre-cancerous. After excision, the specimen often rolls up if immediately put in formalin, making orientation difficult. This can be counteracted by first placing the specimen on sterile suture packing foam for a few minutes to stiffen before putting it in formalin. Fragmentation of small tumour specimens and shearing of the surface layers may occur during processing, making the judgement of depth of involvement difficult.

**Figure 2.**
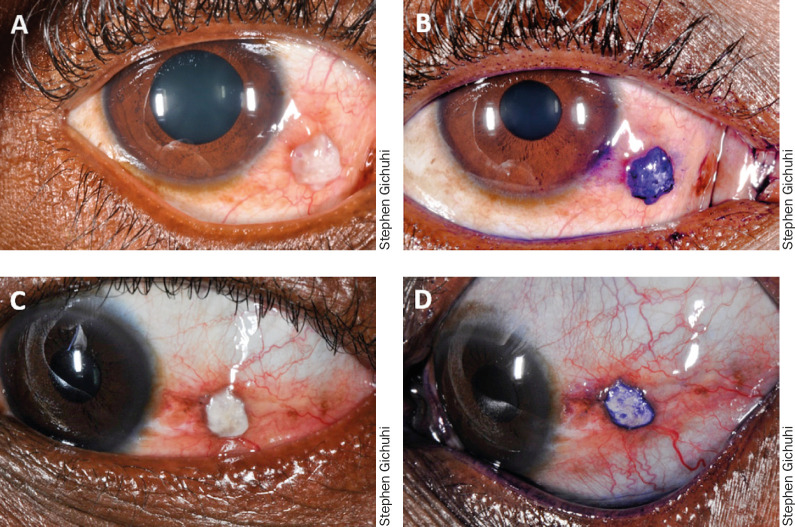
Conjunctival lesions before and after staining with 0.05% toluidine blue. The pictures in the left column are before staining and those on the right after staining. Images A and B show moderately differentiated squamous cell carcinoma, with deep royal blue staining. C and D show actinic keratosis, with mixed staining (margin and parts of the lesion).

Although vital staining with topical toluidine blue 0.05% stains most lesions dark royal blue with a high sensitivity, the specificity is low due to false positives in benign lesions (Figure 2).^4^

## Treatment

Surgical excision under the microscope is the most commonly used technique. Small lesions are simply excised in total while larger ones involving the orbit may need exenteration, a radical technique that involves removing all the orbital contents including the periosteum.

Lesions are excised with a 4 mm margin, dissecting down to the sciera without touching the tumour. Some surgeons use the bare sciera technique which allows the conjunctiva to re-epithelialise, whereas others mobilise the surrounding conjunctiva for primary closure of the defect and earlier post-operative adjuvant chemotherapy. Other ways of closing the defect are by autologous conjunctival graft from the other eye or by using commercially available amniotic membrane. Absolute alcohol is applied to the corneal extension of the lesion to loosen the tissue from the cornea, so that it can be dissected microsurgically with a blade.

Adjuvant therapies to augment surgery include cryotherapy, where 2–4 freeze-thaw cycles are used to obliterate residual tumour at the bed and margins. Topical cytotoxic drugs, such as 5-fluorouracil (5FU) and mitomycin C, may be applied to the bed for about 2.5 minutes then washed off. Other agents include interferon alpha 2b drops, cyclosporin A, all-trans retinoic acid, anti-VEGF agents and radiotherapy. Many centres in Africa do not have cryotherapy or other adjuvants, except for 5FU, which is frequently available. Topical antibiotic-steroid combination eyedrops are applied 4 times daily for about 3–4 weeks after the primary excision, until the site heals.

Recurrence after the primary excision can be frequent. Surgical excision alone is associated with recurrences of 3.2% to 67% at an average of 32 months. HIV testing and treatment should be considered standard practice for all patients presenting with OSSN. We recently conducted a randomised controlled trial of topical 5FU 1% eye drops applied 4 times daily after the excision site healed (usually 2–3 weeks after excision) for OSSN lesions <2 quadrants in diameter.^5^ It decreased the risk of recurrence one year after excision from 36% to 11%. There were transient adverse effects such as a watery eye, discomfort when applying the drops and eyelid inflammation, which settled within 2–3 weeks after completion of treatment. In Kenya the estimated cost of a 4-week treatment course of 5FU eyedrops is 320 Kenyan shillings (US $3.20).

**Figure 3.**
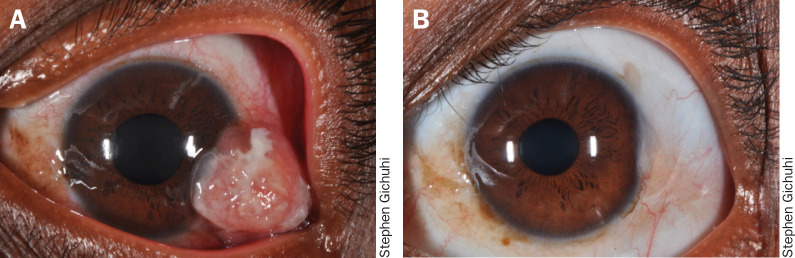
Picture A shows the pre-operative appearance of a lesion in a 32-year-old woman. She was HIV infected with a CD4 count of 69 cellsμ/L. The lesion was excised with a 4 mm margin. She was given topical Gentamycin and Prednisolone drops 4 times daily for 3 weeks. Histopathology showed moderately differentiated squamous cell carcinoma. She was given 1% 5FU drops to apply 4 times daily for 4 weeks. (B) shows the eye about a year later; the lesion had not recurred.

## Follow-up

Follow-up is important to monitor for recurrence, including everting the upper eyelid in case of recurrent tumour on the tarsal conjunctiva. Most recurrences in sub-Saharan Africa present early (3 and 6 months later). Reviews in this region should ideally be done 1, 3 and 6 months after surgery. After one year, reviews may be conducted at month 18, 24 and 36 after surgery. For large lesions that need more radical surgery, the follow-up regimes vary. Some surgeons use radiotherapy after surgery.

## Patient counselling

There is no word for OSSN in most local languages. Calm reassurance is needed, especially as this cancer tends not to metastasise and in the majority of cases is not life threatening. Most patients will be anxious when told that they have cancer in their eye. In those living with HIV, this may be compounded by other concerns related to the complications of HIV. For people with large orbital tumours there may be fear of general anaesthesia. The possibility of recurrence and the need to follow up in the clinic is essential.

It is helpful to give patients evidence of the success of surgical excision with adjuvant therapy (for smaller lesions). For example, former patients who are willing to share their experiences with other patients can be very helpful ‘change agents,’ and can reassure and encourage others to come for treatment.
